# Effect of Vital Pulp Therapy Biomaterials on Tooth Discolouration: A Review of the Literature

**DOI:** 10.1155/ijbm/3080084

**Published:** 2025-05-14

**Authors:** Maedeh Gilvari Sarshari, Kiana Shakeri, Ardavan Parhizkar

**Affiliations:** ^1^School of Dentistry, Shahid Beheshti University of Medical Sciences, Tehran 198396-3113, Iran; ^2^Iranian Centre for Endodontic Research, Research Institute for Dental Sciences, Shahid Beheshti University of Medical Sciences, Tehran 198396-3113, Iran; ^3^Department of Endodontics, School of Dentistry, Tehran University of Medical Sciences, Tehran 1439955991, Iran

**Keywords:** calcium silicate–based cements, endodontic biomaterials, regenerative endodontics, tooth discolouration, vital pulp therapy

## Abstract

Tooth discolouration is addressed as a serious consequence of different traumatic injuries as well as an undesirable outcome of various dental and maxillofacial treatments. In addition, the colour changes of teeth are one of the major concerns and chief complaints of dental patients, specifically when the corresponding injuries and/or treatments occur in the aesthetic zone. Endodontic therapy plays a major role in the formation of discolouration inside and outside the involved teeth since the dehydration of dental structure and the use of different biomaterials in Endodontics and related treatments have shown to result in the creation of colour changes in the involved tooth. Vital pulp therapy (VPT) modalities, as ultraconservative approaches in Endodontics, have demonstrated to simplify endodontic procedures and seem to be able to preserve the dental tissue; however, it has been shown that pulp bleeding and (bio)materials used in VPT could cause different degrees of tooth discolouration mainly due to the residual blood products and components within the formula of the (bio)material, penetrating deep into dentinal tubules while changing the colour of the remaining structure. Consequently, when VPT modalities are considered for the treatment of compromised pulp, their pitfalls should be thoroughly elaborated, especially when the aesthetic zone is involved. Therefore, a thorough understanding of potential factors causing tooth discolouration and possible preventive measures in VPT is mandatory to prevent tooth colour changes, particularly in the anterior teeth or sites where aesthetics is of utmost importance.

## 1. Introduction

In the modern era of dental medicine and its state-of-the-art treatments, aesthetics and aesthetic dentistry have been elaborated as one of the most important priorities considered in the corresponding ministrations [1]. Therefore, a number of dental treatments are specially designed to provide desirable outcomes for patients in need of aesthetics, e.g., laminate veneers, ceramic crowns and resin-based dental composite restorations, for obtaining maximum beauty, specifically in the anterior region of the mouth [2, 3]. Nevertheless, discolouration of teeth is reflected as displeasing and in cases, psychologically traumatising [4]. Tooth discolouration can occur following traumatic injuries and different dental treatments, and is seemingly related to the use of inner bleeding and the applied agents, e.g., (bio)materials, drugs and so forth, used in restorative, endodontic and other treatments [5–8].

In present-day Endodontics, various approaches are deliberated for the treatment of the involved pulpal tissue from ultraconservative methods, i.e., vital pulp therapy (VPT), to invasive modalities, i.e., pulpectomy and conventional root canal therapy [9, 10]. Different studies have reported degrees of tooth discolouration following pulpectomy or traditional root canal therapy due to the relatively total loss of tooth moisture, i.e., dehydration, and subsequent disappearance of tooth translucency [11–13]. It has been shown that endodontically treated teeth lose their capability to sense through pulpal connective tissue and, thus, to respond to environmental changes, undermining their ideal structural integrity, although they can still react to their surrounding stimuli due to proprioception from periodontal ligament [14].

VPT is defined as an ultraconservative approach, aiming at the preservation and maintenance of dental pulp connective tissue compromised by different stimuli, e.g., dental caries and trauma [15]. VPT modalities include different treatments from the concepts of step-wise caries removal/excavation, indirect/direct pulp capping to partial and full pulpotomies, aiming at the preservation of pulp vitality [16, 17]. It seems that VPT can offer benefits over conventional root canal treatment (RCT) including the presence of tooth sensitivity, tooth proprioception as well as damping property and limited dentine regeneration [15, 18]. Reparative dentine formation plays an important part in the maturation of immature permanent teeth due to their incomplete apical closure and undeveloped dentinal walls [14]. A number of research studies have reported that acceptable prognosis and long-term tooth survival need favourable crown/root ratio, sufficient dentine thickness and maintained vitality, which seem to be encouraged by VPT [15, 19]. Additionally, conserving the remaining vital pulp using VPT helps reduce the occurrence of apical periodontitis via keeping the pulp vital, preserving the antimicrobial characteristics of the pulp and blocking bacterial invasion/infection [20, 21].

It has been demonstrated that the choice of a suitable biomaterial and its properties can affect the success or failure of VPT [22]. To succeed in VPT, a wide range of (bio)materials, with various present-day approaches [23], have been introduced as a pulp capping protective dressing and/or barrier [24], which ideally should be able to (i) maintain the remaining pulpal vitality, (ii) stimulate hard tissue formation, (iii) have antibacterial activity, (iv) possess sealability, (v) resist forces during placement of the final restoration and masticatory forces, (vi) show radiopacity and (vii) be nonstaining [24, 25].

Although conservative approaches, e.g., VPT, do not harshly affect the tooth structure compared to invasive or aggressive methods, due to the inadequate removal of coronal pulp, presence of blood, remaining pulpal connective tissue as well as flakes of (bio)materials, eventual tooth discolouration could be expected [9, 26]. In the aforementioned circumstances, the remnants may penetrate into dentinal tubules or nest in distant regions and gradually start to change the colour of the affected tooth. Moreover, it has been shown that the erythrocytes present in the remaining pulp or dentinal tubules degrade into haemosiderin, haemin, haematin and haematoidin, releasing iron during haemolysis [27, 28]. Then, the iron is converted to black ferric sulphide as an outcome of its combination with hydrogen sulphide produced by bacteria, causing eventual grey/brown tooth discolouration [29–31]. In addition, it has been reported that the chemical reaction(s) between the above-mentioned substances and tooth organic matrix, e.g., collagen and other proteins, could cause tooth staining [32]. Furthermore, it has been shown that despite no tubular morphology, organic enamel structure at the dentine–enamel junction may play a role in the discolouration process [27]. The absence of a smear layer on dentine could increase the degree of discolouration following the entrapment of blood, its contamination and alteration of dentine permeability [33].

The elements of (bio)materials used, e.g., iron, bismuth, aluminium, magnesium and their oxidative or reductive reactions, may act as sources for tooth discolouration [34, 35]. The aforementioned compounds release free electrons that are excited by oxidising agents and visible spectrum light, exhibiting strong colour change in their oxide forms [36]. In fact, the free released electrons interact with the collagen matrix (or any other organic constituents) of the dental structure through oxidisation, causing compound build-up which can result in the colour changes of affected teeth [37].

In VPT, the applied biomaterial comes in close proximity with the used oxidising agents, e.g., dentinal collagen and sodium hypochlorite residues, often resulting in oxidative or reductive reactions which could be a reason for tooth discolouration [33, 38]. Collagen is the main protein component of the dentine organic matrix [39]. Interaction, i.e., oxidation, of bismuth oxide with amino acids with collagen may lead to the destabilisation of bismuth oxide and, consequently, black/grey colour changes [40].

Additionally, the presence of sodium hypochlorite (NaOCl), as the most frequently used disinfection solution in VPT, and its tendency to crystallise and block dentinal tubules may result in tooth discolouration [41], specifically when it is placed adjacent to a VPT (bio)material [42]. Studies have demonstrated that NaOCl damages the dentinal collagen network, contributes to the opening of tubule's lumen [43], penetrates into the dentine matrix [44] and may stay in the dental structure [45], causing tooth discolouration. Other existing studies on tooth discolouration have revealed colour changes as an undesirable outcome resulting from the combination of NaOCl with calcium silicate–based cements (CSCs) [45, 46]. Due to the capability to absorb and entrap blood components and metabolites, the porosities in employed (bio)materials have been shown to be a cause for colour changes [47]. Besides, the interaction between erythrocytes and incomplete/unset pulp capping (bio)materials may allow the absorption of blood component and haemolysis of erythrocytes from pulp tissue, resulting in discolouration [40].

**Figure 1:**
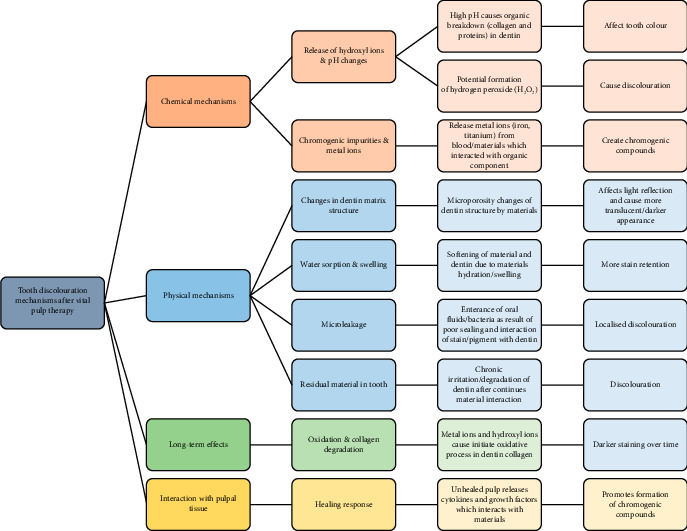
Mechanisms of tooth colour changes after vital pulp therapy.

Chlorhexidine (CHX), which has vastly drawn attention as a substitute for NaOCl [48], is biocompatible and has significant affinity to dentine; however, it has demonstrated an orange-coloured toxic discolouration precipitate if combined with residual NaOCl in dentinal tubules [46]. Research has shown that if CHX is added and mixed with mineral trioxide aggregate (MTA) [49], it could enhance the haemostatic and antimicrobial properties of the cement [50]. Besides, CHX was studied and mixed with CSCs, i.e., white MTA (wMTA), calcium-enriched mixture (CEM) cement and Biodentine (BD), exhibiting highest discolouration [42, 51]. Owing to the presence of bismuth oxide, CSCs may display degrees of discolouration in long term by nature; however, the possibility of discolouration increases when CSCs are contacted with CHX and/or NaOCl [51] (Figure 1).

## 2. Pivotal Biomaterials Used in VPT

### 2.1. Calcium Hydroxide

Calcium hydroxide (CH) has been the most conventional (bio)material used in VPT. It was first introduced to the literature as a root canal filling (bio)material by Hermann in 1920 [15, 52] and was long considered the ‘gold standard' for pulp capping therapy [53]. The first reports of successful pulp healing using CH date back to early twentieth century, i.e., between 1934 and 1941 [54]. Calcium hydroxide has shown to have antibacterial properties, may halt bacterial penetration and can protect pulpal tissue from injuries [15, 55]. Despite the widespread use of CH for pulp closure, investigations suggest undesirable properties for CH, e.g., poor antibacterial activity, formation of porous dentinal bridge, lack of adherence to dentine structure and induction of degeneration/liquefaction necrosis in the superficial layer of pulpal tissue mainly due to CH basicity (∼12.5–13.5) [54, 56, 57]. Furthermore, the solubility of CH in oral fluids may cause inadequate seal against recurrent infections [15, 58]. Based on a recent systematic review and meta-analysis, the success rate of VPT treatments using calcium hydroxide declined from 74% after 6 months to 56% after 4–5 years [59].

There is no strong evidence on discolouration presented by CH [60]; nevertheless, some studies have reported CH-induced change in tooth colour, specifically after modifications made in CH formulation(s) to increase radiopacity and antimicrobial properties [61]. An investigation has demonstrated that the injectable form of CH has shown more staining potential in comparison with its pure form, probably attributed to the presence of bismuth carbonate in the injectable form of CH [62]. However, another study has stated that CH could not induce discolouration [63].

Nowadays, and with the latest advancements in biotechnology, calcium hydroxide is being gradually replaced by new generations of (bio)materials, resulting in more predictable and satisfactory clinical outcomes. CSCs have been widely used as replacements for CH due to their high biocompatibility, intrinsic activity, acceptable osteoconductivity and capability of encouraging regenerative responses, e.g., improved-quality dentinal bridge and high sealability of the pulpal dressed site [54]. In addition, CSCs often exhibit bactericidal and fungicidal properties [64, 65]; however, related studies have reported the exhibition of tooth discolouration as an outcome of CSCs application [33, 66].

### 2.2. Portland Cement

Being the major constituent of MTA, Portland cement has been one of the mostly used endodontic bioactive materials and is deliberated as a CSCs [67, 68]. Portland cement was presented to dentistry in the last century, i.e., early 20^th^, by Josph Aspdin [69], and was frequently considered for use in endodontic treatments due to its antibacterial activity, cell biocompatibility, bio-inductivity, sealability, satisfactory setting time and acceptable physical/mechanical characteristic [68, 70, 71]. Portland cement is mainly composed of tricalcium silicate, dicalcium silicate, tricalcium aluminate and tetracalcium aluminoferrite [68] and contains low amounts of heavy metals (5–100 ppm), i.e., arsenic, chromium and lead [72]. Additionally, the magnesium and iron found in the cement seem to be the cause for its greyish colour [68].

An experimental study has exhibited that Portland cement presents better colour stability amongst cements which are based on Portland; however, the achieved colour stability has not been reported significantly higher than that of wMTA over a period of 1 year [62]. Moreover, it has been stated that Portland cement has the third lowest crown discolouration compared to other (bio)materials, including grey and wMTA [73]. Nonetheless, the addition of bismuth oxide to the composition of the cement as a radiopacifier could result in significant discolouration through various mechanisms. Seemingly, when exposed to temperature, bismuth oxide breaks into bismuth and oxygen, which could eventually result in discolouration following their reaction with pre-existing carbon dioxide in the air [74]. Furthermore, it has been shown that the interaction between bismuth oxide and dentinal collagens can end in dark precipitates [68, 75]. However, upon using zirconia or calcium tungstate as a radiopacifier, higher degrees of colour stability can be achieved [68].

### 2.3. MTA

MTA, as a bioactive endodontic cement, was developed by Torabinejad in 1990s and was then approved by the Food and Drug Administration (FDA) so as to be considered for use in 1997 [76]. It consists of different hydrophilic components, i.e., tricalcium silicate, tricalcium aluminate, dicalcium silicate, tricalcium oxide, bismuth oxide and iron [77]. However, in some formulations, calcium tungstate has replaced bismuth oxide as the radiopacifier [78]. MTA completes its setting process in the presence of water or blood since contact with aforementioned fluids can initiate the formation of crystallised calcium–silicate hydrate gel and calcium hydroxide [77, 79]. Due to its various beneficial characteristics, i.e., biocompatibility [80], bioinductivity [81], bioconductivity [82], sealability [83] and antibacterial activity [84], MTA, as a Portland-based cement, has drawn widespread attention for use in pulpal and periodontal treatments [54, 77, 85]. Moreover, MTA has proven to be more successful than CH in the formation of dentinal bridge [86, 87] which seems to be the result of creating more localised, homogeneous and thicker dentinal tissue with fewer tunnel defects [88], simultaneously causing less inflammatory response and pulp necrosis when compared to CH [89]. Despite its positive properties, MTA comes with shortcomings, including high cost, low flowability, difficult handling, long setting time (2 h 45′) and risk of tooth discolouration [78, 83, 90]. Several studies have demonstrated that the discolouration potential of MTA seems to be the main disadvantage concerning aesthetics [91, 92]. A recent randomised clinical trial found no coronal discolouration following partial pulpotomy of mature permanent teeth with MTA and CH [93]; however, another comparable trial on full pulpotomy in mature permanent molars encouraged the application of BD and TotalFill instead of MTA to reduce discolouration [94].

The first composition of MTA was introduced as grey MTA (gMTA) [80]; however, due to severe discolouration subsequent to its application, the components were altered [95]. Following the removal of tetracalcium aluminoferrite and reduction of the concentration of various metal oxides, the modified cement was introduced as wMTA [53, 80]. Although wMTA presented better characteristics, degrees of discolouration could be still observed [96]. In addition, long setting time of 2 h 45′, low flowability and technique sensitivity were concerning [83, 97].

There are possible mechanisms associated with the discolouration potential of wMTA, which can be elaborated as follows:

i. Oxidation of iron content in wMTA, leading to the formation of calcium aluminoferrite, which is likely to cause tooth discolouration once incorporated into dentinal tubules [98, 99].
ii. Chromogenic changes of bismuth oxide in an oxygen-free environment when exposed to UV light or high temperature, resulting in the formation of metallic black crystals of bismuth, a primary cause of tooth discolouration [100]. In addition, the choice of light source and its features (e.g., wavelength range and brightness) can affect the degree of tooth discolouration induced by wMTA [101, 102].
iii. Chemical reactions between MTA components and phosphate ions or plasma proteins in dentinal fluid, leading to the formation of pigmented products after oxidation [103].
iv. Reaction of bismuth oxide with oxidising agents, e.g., NaOCl, and dentine collagen, forming bismuth carbonate and black precipitates [102]. When bismuth oxide comes in contact with NaOCl, the latter breaks into sodium chloride and oxygen, forming a dark black precipitate through the overoxidation of bismuth oxide [46]. Additionally, the resulted oxygen becomes unstable and, after reacting with the present carbon dioxide in air, forms bismuth carbonate [99].
v. Interaction of bismuth oxide with CHX, although the exact mechanism of tooth colour changes following the use of CHX is not well defined [31].
vi. Contact of MTA with blood components during VPT, which can alter the chromatic effects of the (bio)material used [99]. The amount of blood uptake during setting reaction, presence of porosities and total setting time of the (bio)material can be responsible for the tooth colour alteration. Besides, blood haemolysis can form additional products, e.g., iron, haemoglobin and haematin, that can enter into dentinal tubules and cause discolouration [47].
vii. Increased solubility in wMTA compared to gMTA, which can contribute to tooth discolouration [60].

Consequently, due to the adverse effects of bismuth oxide on tooth colour, several strategies have been proposed to prevent or minimise discolouration, including the placement of MTA below cementoenamel junction [104], addition of zinc oxide or aluminium fluoride to wMTA [105], application of dentine bonding agent prior to MTA application [106] and employment of novel MTA-based (bio)materials (e.g., RetroMTA and Endocem) with radiopacifiers other than bismuth oxide, e.g., zirconium oxide or tantalum oxide [101].

RetroMTA (BioMTA, Seoul, South Korea) is a bioceramic material containing calcium carbonate, silicon dioxide, aluminium oxide and hydraulic calcium zirconia complex [107, 108]. A recent systematic review has claimed that RetroMTA encourages less tooth discolouration [109]. Endocem (Maruchi, Gangwon-do, South Korea) is an MTA-derived pozzolan cement exhibiting comparable biocompatibility, mineralisation potential and expression of odontogenic-related markers to those of ProRoot MTA [107, 110, 111]. In addition, Endocem presents shorter setting time (∼4 min) [112], easier manipulation [113] and higher wash-out resistance [114]. It has been reported that Endocem could cause greyish colour in a short time; however, in the long term, it presented little or no tooth discolouration [79, 115]. Nevertheless, an investigation has claimed that RetroMTA and Endocem induce little to no colour alterations in the tooth structure compared to MTA which contains bismuth oxide [101].

### 2.4. BD

To address and compensate for the limitations of MTA, BD, a tricalcium silicate-based cement (Septodont, Saint-Maur-des-Fossés, France) with seemingly comparable physical properties to dentine, was introduced to Endodontics by Septodont in 2011 [54, 116–118]. The formulation of BD involved alternations in the composition of MTA via incorporating bond accelerators and softeners, so as to enhance its physical properties [119, 120].

BD is available in a single-dose capsule containing a powder and a liquid [121], with (a) the powder composing of tricalcium silicate, tricalcium aluminate, dicalcium silicate, calcium carbonate, calcium oxide, iron oxide and zirconium oxide as radiopacifier, and (b) the liquid consisting of water, modified polycarboxylate and calcium chloride as setting accelerator [122]. BD accelerates apatite formation following its immersion in phosphate solution, which could be an indication of its bioactive nature [123]. Therefore, it is expected to promote dentinal bridge formation similar to MTA [124], a phenomenon that results in increased sealing ability [118, 125]. Furthermore, BD has been shown to have better antimicrobial activity, improved compressive/flexural strength and higher capability to form a good marginal seal in comparison with MTA [54]. Moreover, BD has shown lower setting time and smaller-sized porosity compared to MTA, resulting in the possible blockage of blood component entry, leading to less tooth discolouration [126]. However, a study has reported that BD could be more affected by blood contamination, which may be attributed to the presence of calcium carbonate [47]. In contrast, another investigation has reported no significant difference in tooth discolouration induced by MTA and BD. Seemingly, the type of CSC is not as important as the role of blood in tooth colour alteration(s) [38]. Other research has demonstrated that BD could show acceptable colour stability through preventing blood contamination. A recent randomised clinical trial on VPT using BD and MTA has shown that BD caused no evident discolouration while wMTA demonstrated some degree of discolouration [127]. Another clinical trial on pulpotomy (bio)materials in traumatised anterior immature permanent teeth revealed that despite comparable outcomes of MTA and BD in treatment of immature teeth, tooth discolouration was significantly less when BD was used [128]. In addition, due to its translucency, BD seems to present better colour similarity with natural tooth structure [129].

Zirconium oxide, i.e., zirconia, has recently replaced Bismuth oxide as a radiopacifier to reduce the discolouration potential of BD [130]. In addition, zirconia has favourable mechanical properties, less corrosion resistance and does not affect the hydration of the material [131, 132]. However, the radiopacity of zirconia is significantly lower than that of bismuth oxide and gradually decreases over time [126].

### 2.5. BioAggregate™

BioAggregate™ is a novel white nanoparticle bioceramic material manufactured by ‘Innovative Bioceramix, Vancouver, BC, Canada' and ‘Diadent (i.e., Diaroot)' in 2000s [133, 134] and is considered for use in different endodontic procedures, e.g., pulp capping [135, 136], and consists of tricalcium silicate, dicalcium silicate, calcium phosphate monobasic, amorphous silicon dioxide and tantalum pentoxide as radiopacifier [136, 137]. BioAggregate™ has presented comparable biocompatibility [138], satisfactory sealing ability [139] and notable antibacterial properties [140] when compared to MTA [141]; nevertheless, it comparably shows superior potential to stimulate the formation of mineralised tissue due to its phosphate ion (P_i_) content [142–144]. BioAggregate™ takes approximately up to 4 h to set, presenting a clinical challenge specifically when the immediate placement of final restoration is simultaneously required [145].

BioAggregate™ lacks aluminium and bismuth oxide in its content [146], causing the material to be more resistance to colour changes when compared to wMTA [135]. However, BioAggregate™ exhibits greater porosity and a higher fluid absorption capacity in comparison with BD [147]. The higher fluid, i.e., blood, uptake can be attributed to an increased risk of tooth discolouration in BioAggregate™ in contrast to BD [137]. An experimental investigation on bovine anterior teeth revealed that BioAggregate™ caused one of the highest levels of discolouration in the first year, surpassed only by the effects of the blood control group [148]. However, there are not much data available on the colour stability of BioAggregate™, and thus, the material should be studied further in detail.

### 2.6. CEM Cement

CEM cement is deliberated as a hydrophilic endodontic biomaterial and was first introduced to dentistry as an endodontic filling (bio)material by Asgary et al. in 2006 [71, 149, 150]. It has demonstrated comparable clinical applications to MTA; however, there are several different components [83]. CEM cement is an amalgam of calcium hydroxide, calcium oxide, calcium sulphate, calcium phosphate, calcium carbonate and calcium silicate [141, 151]. The cement releases calcium and phosphate ions, resulting in the formation of hydroxyapatite-like layer in the simulated body tissue fluid and/or normal saline solution [152]. The high pH value (pH = ∼11-12), acceptable biocompatibility and appropriate sealability of CEM cement are similar to MTA [83]; however, other studies have reported that CEM alkalinity is higher than that of MTA, and thus, it is claimed to have superior antimicrobial properties [83, 150, 152]. Furthermore, CEM cement can induce differentiation in human dental pulp stem cells [153].

The antibacterial properties of CEM are similar to CH [154]; nevertheless, its biocompatibility and potential in hard tissue formation following VPT are significantly higher than those of CH [71]. Also, CEM cement has been shown to possess other beneficial features, e.g., shorter setting time [155], greater flowability and less film thickness [141, 152], as well as fungicidal properties against *Candida albicans* even in low concentration [156].

CEM cement lacks iron and bismuth oxide in its content [97] and has less amounts of other oxides, e.g., titanium oxide, aluminium oxide and magnesium oxide [157, 158]. Consequently, it is expected to show less tooth discolouration [97, 99, 115, 157], and thus, the use of CEM cement could be theoretically more recommended in aesthetically involved teeth. Despite the above, previous investigations addressing the incidence of dental discolouration as the outcome of using CEM cement have reported controversial results [159]. A study evaluated the colour stability of wMTA and CEM cement and found that wMTA exhibited less colour stability compared with CEM cement samples [160]. In another study, CEM cement showed no tooth colour alteration compared to MTA [115]. An in vitro evaluation on MTA, CEM and BD showed coronal discolouration following the use of the mentioned (bio)materials; however, the tested CSCs were not significantly different in terms of coronal colour alterations [161]. Nonetheless, it has been reported that CEM cement may induce tooth discolouration in the long term because of the infiltration of colouring agents into the dentinal tubules [158]. The exact mechanism of tooth discolouration induced by CEM cement has not been fully explored; nevertheless, it might be related to its chemical component [162].

### 2.7. EndoSequence® Root Repair Material (ERRM)

In 2014, Brasseler (Savannah, GA, USA) presented ERRM as a premixed bioceramic material [163], potentially used for pulp capping [164], with similar biocompatibility [165], mechanical features [166] and antifungal/antimicrobial properties to MTA [167]. However, a study has reported the faster and higher quality induction of tertiary dentine formation by ERRM compared to that of MTA [168]. ERRM mainly comprises tricalcium silicate, dicalcium silicate, monobasic calcium phosphate, zirconium oxide, tantalum oxide, thickening agents and proprietary fillers; however, no trace of aluminium can be seen in ERRM [169–171]. ERRM is available in two forms, e.g., premixed paste (iRoot BP [Innovative BioCeramix]) and mouldable/condensable putty (iRoot BP Plus [Innovative BioCeramix], Vancouver, BC) [172].

The setting reaction of ERRM begins with the presence of moisture [173], and ERRM shows antibacterial properties during its setting reaction due to high alkaline pH value (> 12) [168, 174]. In addition, the working time is longer (up to 30 min) than that of MTA (5–15 min); nevertheless, it has a shorter setting time (∼4 h) compared to MTA (4–6 h) [173, 175].

Research has demonstrated that ERRM can show favourable colour stability due to the presence of zirconium oxide, instead of bismuth oxide, as a radiopacifier, and, thus, does not induce black discolouration when in contact with dentinal collagen [163, 176]. It has been reported that ERRM could show significantly lower discolouration compared to MTA, primarily due to the existing bismuth oxide in MTA [91]. Moreover, studies have shown that ERRM presents better colour stability and can be considered as an alternative to MTA in the aesthetic zone [163, 177, 178]. However, a single study reported no difference in discolouration induced by MTA and ERRM in the presence of blood, since the blood contamination appeared to significantly increase (bio)material-induced discolouration [73]. On the other hand, Beatty and Svec reported substantially greater colour changes following the use of ERRM in the absence of blood contamination and light irradiation compared to that of MTA [169]. Additionally, an ex vivo study on tooth discolouration following the use of different CSCs has reported that there is no significant difference in causing tooth colour alterations with BD, ERRM and MTA when blood is present. However, when blood is absent, BD and ERRM resulted in less changes in tooth colour compared to MTA [179].

### 2.8. TheraCal LC and TotalFill

Following ∼10-year research, and in 2011, BISCO (Schaumburg, IL, USA) introduced TheraCal LC as a light-curable, biocompatible, single paste resin-modified tricalcium silicate material [180, 181], which could be deemed for use as a pulp capping agent and protective liner in endodontic and restorative dental procedures [117, 182]. It is composed of a hydrophilic resin monomer combined with tricalcium silicate particles. These particles release calcium ions, which stimulate the formation of hydroxyapatite and secondary dentine [47]. In addition, TheraCal LC (TC) exhibits superior flowability, improved sealability and enhanced mechanical strength in comparison with MTA [15, 91, 176]. Moreover, it has been shown that the solubility, porosity and interfacial microleakage of TC are less than those of MTA [182]. TheraCal LC can demonstrate better homogeneity in comparison with other pulp capping (bio)materials, such as BD, which has been reported may entrap blood components within its particles [31]. However, it is important to note that BD has a faster calcium ion–releasing ability compared to TC [183].

The composition of TC includes Type III Portland cement, fumed silica, a resin component and barium sulphate as a radiopacifier with ‘whitish' colour [182]. Consequently, TC may alter the shade of final restoration when it is used under translucent resin-based dental composite restoration [89]; therefore, TC should be used with caution when direct aesthetic outcomes are a concern.

TotalFill is a premixed hydraulic bioceramic material used as pulp capping [184] and is composed of calcium silicate, calcium phosphate monobasic, filler agent, tantalum oxide and zirconium oxide as radiopacifiers [47]. TotalFill has demonstrated acceptable dentinal bridge formation with no pulpal inflammation in VPT owing to its sustained calcium ion release and alkaline pH that can promote pulp regeneration and the formation of a protective mineralised barrier [184, 185]. TotalFill is available in various formulations to accommodate different endodontic applications, such as syringable paste, condensable putty and fast-set putty [186, 187].

The bismuth oxide-containing CSCs can cause tooth discolouration compared with those with other radiopacifier, i.e., zirconium oxide in BD, barium sulphate in TC and tantalum/zirconium oxide in TotalFill. However, a recent study compared coronal discolouration induced by MTA, BD, TC and CEM cement, concluding that all the (bio)materials caused degrees of tooth discolouration despite the absence of iron and bismuth oxide in BD, TC and CEM cement, with TC causing higher colour discolouration [117]. It has been demonstrated that TC can cause less alteration in tooth colour compared to MTA [188], whereas TotalFill and BD do not cause significant colour changes when compared with MTA [189].

In another similar investigation on tooth discolouration, it was demonstrated that TC-induced discolouration could possibly be related to the presence of barium, strontium and zirconium oxide components, with its discolouration potency less than that of MTA [190]. Additionally, another study reported that BD barely caused any discolouration in comparison with TC and TotalFill. It has been also reported that, due to the homogenic and less porous structure, TC and TotalFill are less affected by blood contamination compared to BD [47]. A randomised clinical trial has recommended the application of TotalFill instead of MTA to gain less tooth colour alterations [94].

### 2.9. Clinical Decision-Making: Balancing Aesthetic and Biochemical Considerations

The selection of biomaterials in VPT requires a comprehensive approach that balances aesthetic considerations with the biochemical properties of the (bio)materials. Aesthetic outcomes are predominantly important in anterior teeth, where discolouration can significantly affect patient satisfaction and treatment success. However, (bio)material selection must prioritise the long-term biological efficacy of the procedure, ensuring optimal sealing, biocompatibility and support for pulp healing [191]. Clinicians must assess the needs of each patient (e.g., age, expectations and expense), considering functional and aesthetic factors as well as the specific biological properties of biomaterials available. Such an integrated approach is essential for achieving favourable outcomes while preserving pulp health and appearance of the treated tooth [118, 192]. For instance, MTA is highly effective in sealing and promoting pulp healing; however, it can lead to significant discolouration, particularly in anterior teeth presenting an aesthetic concern. In contrast, CH is less likely to cause discolouration and is often preferred for its aesthetic advantage. Nevertheless, CH is less effective in providing a durable seal and supporting long-term pulp healing compared with MTA. The stated circumstances highlight the need for careful consideration of both aesthetic and biological factors in selecting the most appropriate material for VPT (Table 1).

## 3. Conclusions

VPT is a relatively ultraconservative concept in dental pulp treatment and seems to promote the survival rate of teeth suffering from trauma and deep carious lesions. The preservation of compromised pulp, preparation of a proper matrix for pulpal healing and formation of reparative dentine are elaborated as the primary objectives of VPT. Different biocompatible and bioactive materials, e.g., CSCs, have been introduced to reach the best possible outcome and achieve the aforementioned purposes. However, their adverse effect on the tooth colour is considered a major drawback, and thus, the (bio)material(s) of choice for the VPT modalities should be cautiously selected, specifically when the tooth involved is located in the aesthetic zone.

All CSCs used in VPT can induce degrees of tooth discolouration, and the composition of the (bio)material(s) can be further discussed as one of the main factors responsible for the mentioned drawback. These biomaterials have been shown to promote the remineralisation of tooth structure, which may reduce discolouration but also enhance the long-term integrity of the tooth. The type of radiopacifier, blood contamination, site haemorrhage (lack of haemostasis), technique applied for the treatment and so forth should be considered when VPT is indicated. In addition, a biomimetic approach has been recently introduced to conceivably replicate the natural structure and properties of teeth through mimicking the mechanical properties of dentine and enamel while improving long-term colour stability.

Therefore, future studies could focus on the improvement of traditional materials by optimising their chemical composition and/or the introduction of new formulations with enhanced colour stability (e.g., radiopacifier modification), biocompatibility and pulp regeneration. Furthermore, research could continue to explore the use of nanotechnology, e.g., encapsulating antistaining agents within nanoscale carriers, which could reduce tooth discolouration. Addressing the long-term behaviour of biomaterials, incorporating patient-specific factors, utilising advanced imaging techniques, and exploring innovative strategies to alleviate discolouration can be additionally elaborated. More related studies and investigations, including a methodical systematic review, and well-designed clinical trials are nevertheless needed/necessary to thoroughly evaluate the effects of various biomaterials used in VPT on the discolouration of the dental structure and in hopes of introducing a (bio)materials with the least possible potential for the discolouration of dental structure.

## Figures and Tables

**Table 1:** Summary of (bio)materials used in vital pulp therapy, showing the biological properties, possible clinical indications and limitations of each material, providing a foundation for informed clinical decision-making.

(Bio)material	Biological properties	Clinical indications	Clinical limitations	Aesthetic considerations
Calcium hydroxide	Antibacterial, stimulates reparative dentin, good handling, promotes dentine bridge formation, ability to remineralise carious dentine	Pulp capping (shallow pulp exposures), relieve pain and inflammation in cases with irreversible pulp lesions	Limited long-term sealability, inadequate adhesion to hard tissues, may wash out over time, superficial liquefaction necrosis	Potential for tooth discolouration, especially in injectable form
Portland cement	Antibacterial, biocompatible, promotes dentine formation and pulp healing, bioinductive, high sealing capacity	Root-end filling, rarely used in pulp capping	Cytotoxicity, poor aesthetics	High potential for discolouration
Mineral trioxide aggregate (MTA)	Antibacterial, biocompatible, bioconductive, bioinductive, excellent sealability, promotes pulp healing and promotes mineralised matrix formation	Pulp capping (especially for posterior teeth), root-end filling and endodontic surgery	Difficult to handle, high cost, long setting time, low flowability	High potential of tooth discolouration, particularly in grey form
Biodentine	Antibacterial, superior biocompatibility, improved sealability and better handling compared to MTA, low setting time	Pulp capping (anterior and posterior teeth), pulpotomy	May be more affected by blood contamination, radiopacity, consistent optimisation	Low discolouration potential; limited data on long-term aesthetic outcomes
Bioaggregate	Similar biocompatibility, sealability and antibacterial properties to MTA, good handling	Pulp capping (anterior and posterior teeth), root-end filling	Long setting time	Low discolouration potential; limited data on long-term aesthetic outcomes
Calcium-enriched mixture cement (CEM)	Similar properties to MTA with antimicrobial activity, shorter setting time, fungicidal properties	Pulp capping (anterior and posterior teeth), root-end filling	Long coagulation time (up to 4 h), high material cost	Potential for tooth discolouration, less pronounced than MTA
EndoSequence root repair material	Antibacterial, similar properties to MTA with better tertiary dentine induction	Pulp capping (anterior and posterior teeth)	Long working time, high cost, difficult handling	Relatively favourable colour stability
TheraCal LC	Antibacterial, biocompatible, better mechanical properties and sealability than MTA, good handling, fast setting time	Pulp capping (small pulp exposure), anterior and posterior teeth	‘Whitish' colour	Low discolouration potential, although its whitish appearance can sometimes cause issues under translucent materials
TotalFill	Antibacterial, biocompatible, stimulates dentine bridge formation, fast setting time	Pulp capping (for anterior and posterior teeth), root-end filling	May shrink during setting	Low discolouration potential

## Data Availability

The data used to support the findings of this study are available from the corresponding author upon reasonable request.
